# Narrative Review of the Use of Hydrocolloids in Dermatology: Applications and Benefits

**DOI:** 10.3390/jcm14041345

**Published:** 2025-02-18

**Authors:** Nhi Nguyen, Ajay S. Dulai, Sarah Adnan, Zill-e-huma Khan, Raja K. Sivamani

**Affiliations:** 1 Integrative Research Institute, 4825 J St., Sacramento, CA 95819, USA; 2 Integrative Skin Science and Research, 1495 River Park Drive, Sacramento, CA 95819, USA; 3 Pacific Skin Institute, 1495 River Park Dr Suite 200, Sacramento, CA 95815, USA; 4 College of Medicine, California Northstate University, Elk Grove, CA 95757, USA; 5 Department of Dermatology, University of California-Davis, Sacramento, CA 95816, USA

**Keywords:** hydrocolloid, hydrocolloid dressings, wounds, acne, scar, dermatitis, dermatology

## Abstract

**Background/Objectives:** Hydrocolloid dressings are commonly used in the treatment of chronic wounds by forming a gel-like protective layer upon the dispersion of water, absorbing exudate, and creating a moist environment that promotes healing. However, the use of hydrocolloids has expanded outside of wound care, and this review summarizes the evidence for their use within dermatology. **Methods:** To perform this narrative review, several databases were searched for manuscripts that described the use of hydrocolloid dressings within dermatology. **Results:** The hydrophilic and colloidal dispersion properties of hydrocolloid dressings facilitate the formation of an absorptive, hydrating, and protective layer. In addition, the viscous layer supports innate immunity by activating immune cells such as granulocytes and monocytes, making them effective in wound care. Hydrocolloid dressings appear to be an effective treatment in acute wounds, with the potential of reduced healing time and easier application compared to traditional dressings. The majority of the related research suggests that hydrocolloid dressings and standard dressings have similar efficacy in healing pressure ulcers, and the prevention of hypertrophic and keloid scars. Early reports suggest that hydrocolloid dressings have a role in the treatment of facial dermatitis and acne vulgaris. **Conclusions:** Hydrocolloid dressings have been studied most extensively for chronic wounds and then for use in acute wounds. There have been a few studies on their use for treating acne, facial atopic dermatitis, and hypertrophic scarring. While more clinical studies are needed, there appears to be early evidence of hydrocolloid dressing use within dermatology.

## 1. Introduction

**Figure 1:**
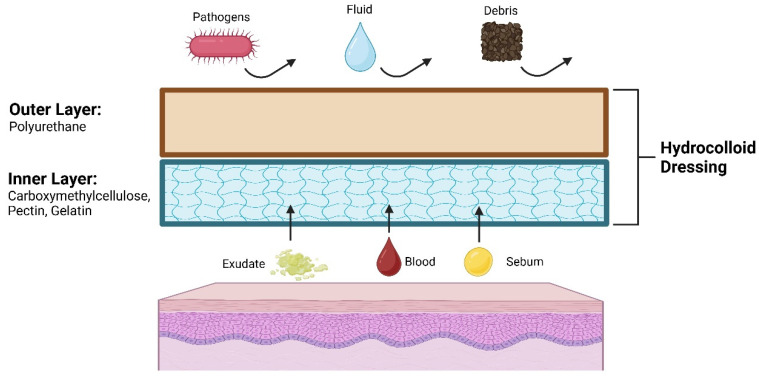
Schematic of hydrocolloid dressing structure and function.

Hydrocolloid dressings (HDs) are widely recognized for their effectiveness in the treatment of various wound types, including both chronic and acute wounds [1]. They are composed of an inner layer which absorbs fluids from wounds and an outer layer which shields and protects the area (Figure 1). Although widely used within the wound healing space, there has been increasing interest in the application of hydrocolloids as small patches for the use in other conditions such as acne [2]. This review summarizes the current indications of hydrocolloid dressing usage and evaluates the current literature for applications within dermatology [3,4,5,6].

Hydrocolloids have been on the market in the UK since 1982, being introduced in the USA in 1983 [7]. Some commercially available hydrocolloid dressings include Duoderm, Aquacel, Comfeel, Atrauman, Mepilex, Tegaderm, Hydrocoll, Allevyn, Systagenix, OpSite, Cutimed, Revita, and Sorbion. The diverse applications of hydrocolloid in the medical and food industries have contributed to their estimated global market value of USD 11.2 billion in 2023 [8].

The term hydrocolloid refers to its composition as a heterogeneous group of long-chain polymers comprising polysaccharides and proteins and characterized by its ability to form viscous dispersions or gels upon contact with water. This occurs when polymer chains cross-link to form a three-dimensional network that immobilizes water, creating a rigid structure resistant to flow and exhibiting properties of both a liquid and a solid. In addition to colloidal dispersion, hydrocolloid is hydrophilic due to its many hydroxyl groups [9]. This property of hydrocolloid allows it to provide a hydrating layer of protection over the site while absorbing exudative fluids [7].

## 2. Materials and Methods

In this review, we aim to assess the evidence on the use of HDs in the treatment of both chronic and acute wounds. In addition, we aim to discuss the mechanism through which HDs exert their benefits in various dermatological conditions. A total of 18 research studies were summarized and discussed in this review. Inclusion criteria consist of English-language research on hydrocolloids, while non-English language studies were excluded unless an English version was available. A literature search on PubMed, MEDLINE, and Embase databases using the following keywords: “hydrocolloid” AND (“wound care” OR “acne” OR “burns” OR “surgical wound” OR “scars” OR “chronic wounds” OR “acute wounds” OR “atopic dermatitis” OR “dermatology”).

## 3. Summary of Findings

All included studies have been summarized in Table 1.

### 3.1. Chronic Wounds

Chronic wounds refer to wounds with a prolonged healing process that extends beyond the expected time frame [28]. In the management of chronic wounds, HDs are an established choice due to their efficacy and durability [7,29,30,31]. A meta-analysis conducted in 2023 assessed 25 studies that contained various dressings and found that HDs had a higher closure rate and the quickest healing time compared to other moist dressings [32]. It is worth noting that although HDs are effective in moderately exudative wounds, they are limited in minimally or highly exudative wounds [7]. Furthermore, HDs are most effective for partial or full-thickness wounds and may be left in place for up to 7 days [33]. HDs consist of two layers: the inner and outer layer. The inner layer consists of a hydrocolloid adhesive that forms a gel over the wound, absorbing exudate and maintaining a moist environment to promote healing. This layer contains hydrocolloid materials such as carboxymethylcellulose, pectin, and gelatin. The outer layer comprises film, foam, or a combination to form a protective layer over the wound and protect it from foreign debris and contamination [29].

However, hydrocolloid dressings may not be effective in all chronic wounds. For example, one study in venous leg ulcerations showed that the use of hydrocolloid dressings was of no benefit when added to compression dressings, suggesting that hydrocolloid dressings may not be of additional benefit in leg ulcers that are already undergoing compression [20].

#### Ulcerative Conditions

HDs are often recognized for their efficacy in the management of ulcerative conditions. A meta-analysis of randomized controlled trials in 2015 comparing treatments with HDs and saline gauze for pressure ulcers found that the probability of complete healing when using HDs increased by over two-fold compared to a saline gauze dressing [34]. Another meta-analysis conducted in 2024 evaluating HDs in postoperative wound healing from maxillofacial surgery suggests that HDs support wound healing and prevent facial pressure ulcers by creating protection and an optimal moisture environment for the wound [35].

A study in 2004 comparing the therapeutic effects of HDs, phenytoin, and simple dressings in 83 paraplegic male victims of the Iran–Iraq war (with participants exhibiting 91 stage I and stage II pressure ulcers) found that HDs were the most effective in facilitating complete healing [19]. The findings from the study suggest that in addition to the absorption of exudate, the viscous layer of an HD allows it to stimulate the immune system by activating granulocytes, monocytes, and the complement system to decrease the risk of infection from bacterial colonization and promote the auto-debridement of the ulcer.

Recent evidence suggests that the efficacy of HDs is due to their ability to create a moist environment for the wound to heal [36]. Notably, the moist environment promotes autolytic debridement, collagen synthesis, and the migration of keratinocytes along the wound surface. Furthermore, the increased humidity facilitates the function of growth factors in the wound microenvironment [37,38]. In addition to facilitating in angiogenesis and granulation, it also causes the pH of the wound surface to decrease to create an acidic environment that inhibits bacteria growth [39].

Despite evidence supporting its effectiveness in pressure ulcers, HDs do not appear to be more effective than other types of dressing in promoting the healing of diabetic foot ulcers [15,16,17,18,40,41]. A 24-week study comparing an HD with non-adherent, knitted, viscose filament gauze and Inadine in individuals with type I or type II diabetes with chronic full-thickness foot ulcers found that all three dressings had no difference in effectiveness [15]. The average proportion of ulcers healed in the HD group and Inadine group was 45% and 44%, respectively.

Another study in 2007 compared an HD containing antimicrobial (ionic silver) with Algosteril calcium alginate (CA) dressings in out-patients with type I or type II diabetes and non-ischemic Wagner Grade 1 or 2 diabetic foot ulcers. The results concluded that there were no significant differences in the healing times between the two groups. However, it was noted that there was an increase in the overall ulcer improvement and less deterioration in the HD group compared to the CA group [16].

In a different 2-week study, Kuo compared an HD with a topical containing Plectranthus amboinicus (*P. amboinicus*) and Centella asiatica (*C. asiatica*) in 24 diabetic participants with Wagner stage 3 ulcers. Although the results from the study did not report the number of ulcers healed, the wound size in both groups were not statistically significant [17].

The above findings are consistent with a 2001 study by Piaggesi where 20 participants with type I or type II diabetes and foot ulcers measuring 1 cm deep or more over the course of 3 weeks were enrolled. Participants were randomized to apply an HD or saline-moistened gauze, and it was found that there was no statistical significance in the number of ulcers healed between the two groups [18].

There is evidence suggesting that this is due to the occlusive nature of HDs, and a study advised against using HDs when the surrounding skin is infected, as can more often be the case of diabetic foot ulcerations that may have long-standing trauma and underlying necrosis [39].

### 3.2. Acute Wounds

Acute wounds refer to a disruption in the integrity of the skin with a predictable healing time frame and progression [42]. Although most of the studies involving HDs are focused on chronic wounds, emerging evidence suggests that HDs also provide advantages in the treatment of acute wounds [6,7]. In 2020, 8-week-old diabetic male mice were inflicted with wounds to the back with full-thickness skin defects and covered with HDs and hydrogel dressings or gauze (control). This study found an increase in M1 macrophages in the early stages of the injury and the subsequent appearance of M2 macrophages in the hydrogel group compared to the control group. Furthermore, the localization of neutrophils and VEGF increased, suggesting that the moist environment provided by the HD with hydrogel improved wound healing compared to the control [26].

Despite the discussed benefits, a study in 1997 comparing two types of HDs (Comfeel and Duoderm) in full-thickness skin wounds of Sprague Dawley male rats addressed components of HDs that may adversely influence wound healing. By day 10, the presence of foam cells indicated that the components from the two dressings were phagocytosed. The extracellular vacuoles occupied around 23.3 ± 14.3% of the granulation tissue volume in the Duoderm group, which is significantly different in comparison to the Comfeel group (4.6 ± 4.5%). This finding is further supported as the Comfeel group were significantly more epithelialized (77.6 ± 23.1%) compared to the Duoderm group (41.3 ± 27.3%). The results from the study indicated that the decrease in epithelialization in the Duoderm group with no change in epithelial proliferation in both groups may be explained by an impaired migratory ability. The findings from the study suggest that HDs with low cohesive matrix cohesion should be replaced by dressings that are more resistant to disintegration when in contact with wounds [27].

#### 3.2.1. Burns

The hydrating and protective effects of HDs have made them particularly useful in the healing of acute wounds. Burn injuries are a result of skin contact with a heat source including high temperature friction, radiation, chemicals, and electricity [43]. Following an acute burn, the disruption of cellular membranes and generation of reactive oxygen species make hypovolemia a potential complication, if not dressed properly [44]. A randomized trial published in 1993 evaluated the Granuflex ‘E’ hydrocolloid dressing and compared its healing to traditional wound dressing when treating partial-thickness burns [23]. The study found that the hydrocolloid dressing resulted in more subjects achieving “excellent” wound healing (50%) when compared to the traditional dressing (11%) (*p* = 0.00099). Notably, they reported that the hydrocolloid dressing had to be changed more frequently due to leakage (16%) compared to the control (3%) (*p* = 0.01). The study found no difference in the time to recovery (median = 12 days, *p* = 0.8897). A similar study compared the hydrocolloid dressing DuoDERM Burn Pack Hydroactive Dressings to conventional silver sulphadiazine/Bactigras dressings in the management of small partial-thickness burns [21]. The study found that the hydrocolloid dressing was easier to apply (*p* = 0.0009) and remove (*p* = 0.0004), and required fewer dressing changes (3 vs. 9; *p* = 0.0117). Similarly, the study found no significant difference in the healing time with a mean of 11 days. In the study, two patients in the hydrocolloid group had a wound infection and one in the control group.

The usage of hydrocolloid materials warrants investigation into the potential infection risk. A study compared the Granuflex hydrocolloid patch against a chlorhexidine tulle gras dressing and the combination of a hydrocolloid patch and an anti-bacterial cream (silver sulphadiazine) while swabbing for bacterial growth at the wound site [22]. While the chlorhexidine tulle gras group had the least increase in overall bacteria compared to both of the other groups (*p* < 0.01), there was no significant difference in the increase in pathogenic bacteria in all three groups (*p* = 0.12). The study suggests that the usage of hydrocolloid patches does not increase the presence of pathogenic bacteria, but wound care and regular dressing changes are still required to avoid infection, similarly to other dressings.

#### 3.2.2. Donor Sites

Similarly to burn sites, skin graft donor sites require adequate dressing to support healing. One study evaluated the Granuflex/Duoderm hydrocolloid dressing and compared its efficacy to paraffin gauze. The study, involving, 24 patients found that the hydrocolloid dressing exhibited a faster healing time (6.8 days) compared to control (10.4 days) (*p* < 0.01) [24]. These results were further supported by other studies with similar results. A randomized controlled trial of 60 subjects compared the healing time, comfort, convenience, infection risk, and cost of using an HD (DuoDerm E^R^) with a gauze containing 5% o-tolylazo-o-tolylazo-beta-napthol, lanolin, and olive oil (Scarlet red) [25]. After assessing the donor site at the 10th postoperative day, the researchers reported that significantly more subjects in the HD group achieved complete healing (90%) compared to the gauze group (57%) (*p* < 0.01). Subjects reported that the HD had less intense and less pain (*p* < 0.05). The HD group had an average of 0.8 leakages per donor site, and the gauze group had 0.04 leakages per donor site. Additionally, neither group experienced an infection. However, the average cost of the HD (NZD 47.54) was more than that of the gauze (NZD 6.27).

#### 3.2.3. Surgical Wounds

Surgical wounds are incisions made in the skin during a surgical procedure [45]. Hydrocolloids have also been investigated for their efficacy in the secondary intention healing of acute surgical wounds with mixed results. One study found that the usage of hydrocolloids following the excision of pilonidal sinuses compared to conventional gauze did not result in a faster healing time (*p* > 0.05) [46]. However, the study did report that hydrocolloid application resulted in significantly less pain in the first four postoperative weeks (*p* < 0.05). However, another study comparing Granuflex/Duoderm with traditional dressing (hypochlorite irrigation and packing with paraffin gauze) found an average healing time of 6 weeks in the hydrocolloid versus 10 weeks in the control [47].

### 3.3. Applications in Dermatology

#### 3.3.1. Acne Vulgaris

One clinical trial evaluated the therapeutic benefits of HDs for the treatment of acne in a pilot study of 20 patients with mild to moderate acne vulgaris. A significant reduction in the severity of acne and inflammation was observed in the HD group. In addition, it is reported that the ratio of the transmission of UVB light reaching the skin surface in the HD group was less than the control group, suggesting that the HDs had a photoprotective effect [11].

A conference abstract described a 14-day randomized, controlled study that investigated the efficacy and tolerability of HDs compared to gentle washing with no treatment in individuals aged 12–35 with at least two inflammatory lesions, one of which was capable of being extracted [2]. The results demonstrated a significant improvement in the texture, erythema, size, and elevation of the extracted pimple at different time points when they were treated with HDs and gentle washing compared to only gentle washing. The investigators concluded that HDs are an effective and rapid option for the treatment of acne. The fully peer-reviewed manuscript was not available at the time of this review.

Interestingly, despite the growing popularity and demand among consumers for HDs in the form of patches, there is limited published research on the direct benefits of HDs in the treatment of acne vulgaris. The mechanism of action may be related to the ability to absorb exudative material, based on its actions in wound healing, but this requires further clinical studies on the use of HDs for acne. Another consideration is the use of medical-grade patches with added compounds like benzoyl peroxide to inhibit bacterial growth [48], although there are currently no studies suggesting that the addition of other compounds to HDs would be beneficial.

#### 3.3.2. Dermatitis

In prevalent dermatological conditions like atopic dermatitis, where pruritus and subsequent uncontrollable scratching significantly impact patient outcomes, established treatments such as wet wraps and other bandaging techniques can be challenging to administer, particularly in pediatric patients [10].

Rademaker investigated the use of a face-masks using HDs in three pediatric patients with recalcitrant facial atopic dermatitis [10]. The 2-week study found that there was a considerable improvement in symptoms of pruritus and soreness by day 8. In addition, all three patients reported longer remissions of their facial eczema despite continued eczema on their trunk and limbs. The findings suggests the potential for the therapeutic benefits of HDs in atopic dermatitis in pediatric patients.

Interestingly, there are several case reports in the past decade that documented sensitization to HDs resulting in contact dermatitis. In a 1997 case report, three patients developed eczematous lesions under HDs [49]. The authors suggested that this was due to the HDs containing the pentaerythritol ester of hydrogenated rosin as the tackifying agent, which retains the sensitizing potential of colophony, a highly potent allergen. In another case report by Kober, which supports these findings, a 62-year-old female patient with a venous leg ulcer who used an HD developed contact dermatitis. In addition, the author conducted a literature review and discovered multiple similar cases with identical presentations but distributed under different brand names [50].

#### 3.3.3. Hypertrophic Scars and Keloids

HDs are self-adhesive and have the capability to be used as primary or secondary dressings. Emerging evidence supports the use of HDs for the treatment of hypertrophic scars [51]. In 2001, a randomized controlled trial compared a silicone gel dressing and HD in 41 keloids and hypertrophic scars present in 26 patients. By 4.5 months, the study found that the HD was equally as effective as the silicone gel dressing in significantly reducing scar size, pigment, and symptoms [12]. The findings demonstrated that the HD exhibited comparable efficacy with the added advantage of self-adhering to difficult-to-reach areas on the body and to body regions with greater mobility as opposed to the silicone gel dressing. This was further supported in 2020 when Oliveira et al. presented two separate case reports in which an HD improved symptoms of pain, pruritus, and texture in the hypertrophic scars or keloids after 3–6 weeks [51].

A 1996 study evaluated keloids and hypertrophic scars in 20 patients, in which they were randomized into the HD group or moisturizer (control) group over a 2-month period. Individuals in the HD group had the dressing applied for up to 7 days and replaced if it became dislodged, while the moisturizer was applied to individuals in the control group once daily. The results found a significant reduction in symptoms such as pruritus and pain with an increase in pliability in both groups, suggesting that the hydration environment provided an improvement of symptoms. It is proposed that the occlusive dressing facilitates the accumulation of growth factors [13].

Additionally, a study assessing the effect of an HD on the hypertrophic scarring of post-cesarean section (C/S) wounds found preventative benefits. The study randomized 135 patients that underwent C/S at the same institution, in which 47 were placed in the intervention group and 24 in the control group. The findings from the study concluded that applying the HD on postoperative day 7 or 8 and continued weekly dressing changes over the course of 6 months reduces the risk of hypertrophic scarring after C/S [14].

## 4. Conclusions

The use of HDs is an established form of treatment for chronic wound care due to their hydrophilic and colloidal dispersion properties [9]. By forming a protective gel layer and absorbing exudate, HDs are particularly efficacious in wound healing. Furthermore, similar benefits are observed in acute conditions like burns and surgical wounds. Although there is growing popularity and consumer demand for hydrocolloid patches in acne, there are few clinical studies available to definitively support their efficacy. Nevertheless, early research reports in the literature suggest that the absorptive nature of the dressing may also be beneficial in the acute lesions of acne that may be exudative. There are no true contraindications to the usage of hydrocolloid dressings; however, we recommend caution in using these dressings in individuals prone to anaerobic infections and those with hypersensitivities to the dressing materials [1]. Also, people with sensitive or thin skin should be careful with the repeated application and removal of hydrocolloid dressings as repeated removals may disrupt the stratum corneum [52]. Future research should investigate the efficacy of hydrocolloid dressings through controlled clinical trials to provide more evidence regarding their direct impact on the treatment of acne. Studies exploring and comparing the many available types of HDs on the market to assess for efficacy and safety are also warranted, especially comparative studies assessing unmedicated vs. medicated hydrocolloid patches, as it is not clear whether the addition of medications will be helpful. Finally, there is early support for the use of hydrocolloid patches for the control of facial involvement in atopic dermatitis and for use in hypertrophic scarring. Larger clinical studies will help to elucidate the clinical evidence further and provide a better understanding of the mechanism of action.

## Figures and Tables

**Table 1:** Summary of findings.

Author (Year)	Indications	Intervention	Study Groups	Results
**Dermatological Conditions**				
Rademaker (2013) [10]	Facial Dermatitis	DuoDerm extra-thin dressings/face-masks; 2 weeks	Three pediatric subjects ages 3, 4, and 4.5 with atopic eczema from 6 to 9 months of age	All subjects reported improvement by day 7 with longer remissions of facial eczema
Chao (2006) [11]	Acne Vulgaris	Acne dressing with HD, skin tapes (control); 7 days	Subjects with mild to moderate acne vulgaris (*n* = 20)	Significant reduction in overall severity of acne and inflammation from day 3 to 7 in the HD group
Kosmoski (2024) [2]	Acne Vulgaris	Treatment group (gentle wash and hydrocolloid patches), control group (gentle wash only); 14 days	Subjects aged 12–35 with at least 2 inflammatory lesions and 1 capable of being extracted (*n* = 41)	Treatment group had significant improvement in texture, erythema, size, and elevation
**Scars**				
Oliveira (2001) [12]	Hypertrophic or Keloid Scars	Silicone, nonsilicone gel dressing (HD), or none (control group); 4.5 months	26 subjects aged 15 to 53 with hypertrophic or keloid scars (*n* = 41)	Silicone and nonsilicone gel dressings are equally effective in treating keloids and hypertrophic scars
Phillips (1996) [13]	Hypertrophic or Keloid Scars	HD group (dressing placed for up to 7 days at a time) or control group (moisturizer applied once daily); 2 months	Subjects with keloids or hypertrophic scars (*n* = 20)	Both groups reported a significant reduction in pruritus and pain and an increase in pliability
Tsubouchi (2024) [14]	Hypertrophic Scars (Prevention)	HD group applied to postoperative wound on day 7 or 8 and weekly dressing changes for 6 months (*n* + 23), control group refrained from any dressing application (*n* = 24)	Subjects who underwent C/S at the same institution (*n* = 135) in which 47 were included in the analysis	Application of HDs to wounds reduces the risk of hypertrophic scarring after C/S
**Chronic Wounds**				
Jeffcoate (2009) [15]	Diabetic Pressure Ulcer	N-A (non-adherent, knitted, viscose filament gauze) (*n* = 106), Inadine (iodine-impregnated dressing) (*n* = 108), Aquacel (HD) (*n* = 103); 24 weeks	Individuals aged 18 or older with type I or type II diabetes and chronic full-thickness foot ulcer	No statistical significance in the number of ulcers healed between the N-A and the Inadine group.
Jude (2007) [16]	Diabetic Pressure Ulcer	HD with 1.2% ionic silver (Aquacel Ag, ConvaTec) (*n* = 67), Calcium-alginate dressing (Algosteril, S&N Hlth (*n* = 67); 8 weeks	Individuals with Wagner Grade 1 or 2 ulcers	No statistical significance in the number of ulcers healed between the two groups.
Kuo (2012) [17]	Diabetic Pressure Ulcer	HD (Aquacel, ConvaTec) (*n* = 12), topical cream with P. amboinicus and C. asiatica (1.25% active ingredients) (*n* = 12); 2 weeks	Individuals with type I or type II diabetes and Wagner Stage 3 ulcers	No statistical significance in the number of ulcers healed between the two groups.
Piaggesi (2001) [18]	Diabetic Pressure Ulcer	HD (Aquacel, ConvaTec) (*n* = 10), Saline-moistened gauze (*n* = 10); maximum follow-up is 350 days	Individuals with type I or type II diabetes and foot ulcer deeper than 1 cm for over 3 weeks	No statistical significance in the number of ulcers healed between the two groups.
Hollisaz (2004) [19]	Non-diabetic Pressure Ulcer	HD, phenytoin cream (PC), simple dressing (SD); 8 weeks	Paraplegic males (*n* = 83) with stage I and stage II pressure ulcers (*n* = 91)	HD has superior efficacy in treating pressure ulcers compared to PC and SD
Backhouse (1987) [20]	Venous Leg Ulcer	HD (Granuflex) with graduated compression bandage vs. porous non-adherent dressing with graduated compression bandage	Patients with venous ulcers (*n* = 56)	No statistically significant difference between the hydrocolloid and the porous non-adherent dressing groups.
**Acute Wounds**				
Afilalo (1992) [21]	Burn Wounds	Hydroactive Dressing (DHD) (*n* = 15) and Bactigras (SSD) group (*n* = 15)	Subjects with partial skin thickness burns with less than 15% TBSA and less than 48 h old (*n* = 30)	Both groups were equivalent in comfort and wound healing; DHD was easier to apply; SSD was easier to remove
Thomas (1995) [22]	Burn Wounds	Chlorhexidine (*n* = 18), hydrocolloid-only (*n* = 16), hydrocolloid and anti-bacterial cream (*n* = 16)	Subjects with less than 5% body surface area burns (*n* = 50); 54 burn sites observed	Hydrocolloid-only group had a shorter healing period and required fewer dressing
Wright (1993) [23]	Burn Wounds	Granuflex ‘E’ (HD) (*n* = 49) and Bactigras (*n* = 49)	Male and female (*n* = 98) with partial skin thickness burns	Granuflex ‘E’ group is safe and effective in the treatment of partial skin thickness burns
Champsaur (1986) [24]	Surgical Wounds	Granuflex/Duoderm hydrocolloid, paraffin gauze (control)	Subject with virtually symmetrical donor sites (*n* = 20)	Hydrocolloid group had a faster healing time compared to control group
Tan (1993) [25]	Donor Site	DuoDerm E^R^ (*n* = 30), Scarlet Red (*n* = 30)	Subjects with single donor site on inner arm or inner thigh (*n* = 60)	HD, which had more subjects with complete healing on 10th day postoperative
Takeuchi (2020) [26]	Wound (mice)	Full-thickness skin defects were inflicted on the backs of diabetic mice and covered with HD with hydrogel or gauze (control); 2 weeks	8-week-old diabetic male mice (C57BLKS/J Air)(db/db) and 8-week-old control mice (C57BL/6JJmsSlc)	Increase angiogenesis in wounds, macrophage polarization to M2 phenotype, and proliferation of fibroblast
Agren (1997) [27]	Wound (rats)	Full-thickness skin defects were inflicted to the thoracic region of rates and treated with Duoderm (ConvaTec), Comfeel (Coloplast A/S), or Adhesive polyurethane film (non-HD control); 10 days	Sprague Dawley male rats weighing 350–550 g (*n* = 14)	Extracellular vacuoles in the Duoderm group occupied more granulation tissue volume. Significant decrease in epithelialization in the Duoderm group with no significant change in epithelial proliferation or wound contraction.

Key: Hydrocolloid dressing (HD), cesarean section (C/S).

## Data Availability

Data are contained within the article.
